# Undetectable equals untransmissible: knowledge, attitude, and practice among HIV-negative clients attending selected health facilities in Rwanda

**DOI:** 10.11604/pamj.2023.44.15.37060

**Published:** 2023-01-06

**Authors:** Pierre Gashema, Zephanie Nzeyimana, Felix Ndahimana, Patrick Gad Iradukunda, Vedaste Masengesho, Elyse Jeanne Umuhire, Olivier Welcome, Muhayipundu Ribakare, Nadine Rujeni, Tafadzwa Dzinamarira

**Affiliations:** 1 College of Medicine and Health Sciences, University of Rwanda, Kigali, Rwanda,; 2 ICAP Columbia University, Kigali, Rwanda,; 3 Department of Drugs and Food Assessment and Registration, Rwanda Food and Drugs Authority, Kigali, Rwanda,; 4 School of Public Health, Mount Kenya University, Kigali, Rwanda,; 5 World Health Organization (WHO), Kigali, Rwanda,; 6 School of Health Systems and Public Health, University of Pretoria, Pretoria, South Africa

**Keywords:** Sustained virologic response, HIV, AIDS, knowledge, attitude

## To the editors of the Pan African Medical Journal

The HIV/AIDS epidemic continues to be an important public health problem globally, particularly in sub-Saharan Africa (SSA) [1]. The unavailability of treatment makes HIV a lifetime viral infection [2]. The discovery of antiretroviral (ARV) regimens has been among the significant achievements in the HIV response with PLHIV now able to easily attain and maintain viral suppression [2]. Rwanda generally experiences good ART coverage, attributable in part, to the test and treat-all PLHIV policy that was initiated in 2016 [3]. According to the 2019 UNAIDS report on the progress of the 90: 90: 90 targets, the country reported 89: 97: 91 for the general population. As with other countries in SSA, Rwanda´s first 90 target remains an important gap [4]. U=U message was beneficial in stigma reduction in entire communities [5]. This approach was launched on 20^th^ July 2018 by UNAIDS and three years later in Rwanda. Given the scarcity of information on the U=U message in SSA including in Rwanda, there is a need to understand the behavioral, and social implications associated with the knowledge of the U=U concept. Thus, we conducted a study to determine the knowledge, attitude, and practices of the HIV-negative clients (based on self-report) attending selected health facilities in Rwanda.

This was a cross-section study that collected data from 380 adults, who are HIV-negative, and who attended selected Health Facilities from May 8^th^- 31^st^, 2022. Our study findings show that 80.8% of the study participants had good knowledge of HIV transmission. The findings further revealed that 6.3%, 5.8%, and 4.7% of the participants had good knowledge, attitudes, and practice toward the U=U phenomenon, respectively further details were described in Table 1.

**Table 1:** factors associated with knowledge, attitude and practice towards U=U among HIV-negative clients attending selected health facilities in Rwanda

		U=U Knowledge			U=U Attitude			U=U Practice		
Characteristics	N	Category		P Value	Category		P Value	Category		P Value
		Good	Bad		Good	Bad		Good	Bad	
**Gender**										
Male	214	14(6.5)	200(93.5)	0.506	14 (6.5)	200(93.5)	0.476	13(6.1)	201(93.9)	0.163
Female	166	10(6.0)	156(94.0)		8(4.8)	158(95.2)		5(3.0)	161(97.0)	
**Age**										
15-24 years	70	1(1.4)	69(98.6)	0.044	2(2.9)	68(97.1)	0.245	2(2.9)	68(97.1)	0.324
25 years and above	310	23(7.4)	287(92.6)		20(6.5)	290(93.5)		16(5.2)	294(94.8)	
**HFs/District**										
Masaka HC	85	4(4.7)	81(95.3)	0.083	3 (3.5)	82(96.5)	0.238	1(1.2)	84(98.8)	0.094
Nyagatare HC	99	2(2)	97(98.0)		3(3.0)	96(97.0)		3(3.0)	96(97.0)	
Remera HC	107	11(10.3)	96(89.7)		9(8.4)	98(91.6)		9(8.4)	98(91.6)	
Rukara HC	89	7(7.9)	82(92.1)		7(6.9)	82(93.1)		5(5.6)	84(94.4)	
**Marital Status**										
Single	150	8(5.3)	142(94.7)	0.304	6(4.0)	144(96.0)	0.642	6(4.0)	144(96.0)	0.361
Married	77	9(11.7)	68(88.3)		7(9.1)	70(90.9)		7(9.1)	70(90.9)	
Cohabitating	49	2(4.1)	47(95.9)		3(6.1)	46(93.9)		2(4.1)	47(95.9)	
Widowed	58	3(5.2)	55(94.8)		3(5.2)	55(94.8)		2(3.4)	56(96.6)	
Divorced	46	2(4.3)	44(95.7)		3(6.5)	43(93.5)		1(2.2)	45(97.8)	
**Relationship duration in years**										
0-3 years	69	3(4.3)	66(95.7)	0.482	4(5.8)	65(94.2)	0.863	1(1.4)	68(98.6)	0.415
4-6 years	40	2(5.0)	38(95.0)		2(5.0)	38(95.0)		3(7.5)	37(92.5)	
5-8 years	43	5(11.6)	38(88.4)		3(7.0)	40(93.0)		3(7.0)	40(93.0)	
9 years +	81	6(7.4)	75(92.6)		7(7.4)	74(91.4)		5(6.2)	76(93.8)	
**Occupation**										
Vocational works	133	8(6.0)	125(94.0)	<0.01	10(7.5)	123(92.5)	<0.01	8(6.0)	125(94.0)	0.086
Healthcare worker	24	10(41.7)	14(58.3)		6(25.0)	18(75.0)		4(16.7)	20(83.3)	
Farming and livestock	40	3(7.5)	37(92.5)		4(10.0)	36(90.0)		2(5.0)	38(95.0)	
Small trade	115	0(0)	115(100)		1(0.9)	114(99.1)		3(2.6)	112(97.4)	
Student	21	0(0)	21(100)		0(0)	21(100)		0(0)	21(100)	
No occupation	8	0(0)	8(100)		0(0)	8(100)		0(0)	8(100)	
Others*	39	3(7.7)	36(92.3)		1(2.6)	38(97.4)		1(2.6)	38(97.4)	
**Education level**										
Primary and below	114	5(4.4)	109(95.6)	0.002	9(7.9)	105(92.1)	0.233	7(6.1)	107(93.9)	0.298
Secondary	151	4(2.6)	147(97.4)		5(3.3)	146(96.7)		4(2.6)	147(97.4)	
University and above	115	15(13.0)	100(87.0)		8(7.0)	107(93.0)		7(6.1)	108(93.9)	
**Area of residence, rural or urban**										
Rural	104	5(4.8)	99(95.2)	0.05	6(5.8)	98(94.2)	0.588	3(2.9)	101(97.1)	0.417
Urban-rural	155	6(3.9)	149(96.1)		7(4.5)	148(95.5)		7(4.5)	148(95.5)	
Urban	121	13(10.7)	108(89.3)		9(9.0)	112(92.6)		8(6.6)	113(93.4)	
**HIV transmission knowledge**										
Good	307	24(7.8)	283(92.2)	0.005	20(6.5)	287(93.5)	0.214	17(5.5)	290(94.5)	0.132
Poor	73	0(0)	73(100)		2(2.7)	71(97.3)		1(1.4)	72(98.6)	
Total, n (%)	380	24(6.3)	356 (97.3)		22(5.8)	358(94.2)		18(4.7)	362(95.3)	

* Other professions included individuals that reported retirement, student, teacher, local leader, bank employees

The WHO and UNAIDS endorsed the U=U phenomenon as the global goal for minimizing stigma and HIV-related discrimination in the surrounding community. However, there have been no studies that have assessed possible deterrents associated with U=U messaging in the Rwandan community.

The present findings indicated that the majority 80.8% of the study participants had good knowledge of HIV transmission. These findings were slightly higher than what was reported from the Rwanda demographic health survey (64%) (DHS, Rwanda 2021). Interestingly, another scholar in Rwanda reported that 99.1% of the study participants had accurate knowledge about modes of HIV transmission [6]. These findings demonstrate the investment that has been put in HIV public health communication in Rwanda.

While HIV transmission knowledge was good among our study participants, KAP scores towards the U=U phenomenon were low. Our study findings revealed a relatively low knowledge towards the U=U, with only 6.3% reporting awareness. In contrast, slightly higher knowledge of HIV naïve individuals about the U=U phenomenon was reported by studies conducted in Brazil, and the USA with 17.2%, and 19% respectively [5]. It is noteworthy that information on U=U has been disseminated more rapidly in PLHIV and key populations, compared to non-HIV individuals or unknown status. This disparity might be attributable to variability in exposure to sexual health information.

In addition, our study showed that only 5.8% of HIV-naïve people had a positive attitude towards U=U. Considering the known positive correlation between knowledge and attitude [7]. It follows that HIV naïve individuals with significantly less knowledge (6.3%) towards U=U could have also a poor attitude towards U=U. Thus, it is incumbent that provision of sustained education on U=U is required to address the poor attitude.

The current data also shows that only 4.7% of HIV naïve respondents had a good practice towards U=U. This skepticism of U=U science and potential behaviors risks were also reported in Kenya and Uganda where HIV-negative partners of people living with HIV reported doubts about the effectiveness of U=U, and in turn preferred to use PrEP or condoms even if their partners were virally suppressed [8]. In contrast, in a survey conducted in the USA, 59% of HIV-negative MSM agreed that they can have safe sexual intercourse without a condom if the partner´s VL is undetectable [9]. This increased practice toward U=U in the USA could be associated with increased knowledge and experience about U=U.

Regarding determinants of good knowledge, attitudes and practice towards U=U, this study revealed that being married, working in the health sector, achieving a higher level of education and living in urban areas predicted an increased level of knowledge, attitude and practice towards U=U. These findings were in agreement with the study conducted in India that reported a higher probability of having inadequate knowledge about HIV among illiterate, individuals from poor households, and rural dwellers [9]. These might be attributable to minimal access to information and/or just ignorance. Therefore, highlighting the need and importance of disseminating HIV-related knowledge among economically and socially disadvantaged communities.

**Conclusion:** the current study found that HIV naïve individuals had a satisfactory level of knowledge on HIV transmission. However, they demonstrated inadequate knowledge of the U=U concept. Therefore, it is imperative to reinforce the existing sexual and HIV/AIDS education as well as public information campaigns about U=U.
